# Dimerisation induced formation of the active site and the identification of three metal sites in EAL-phosphodiesterases

**DOI:** 10.1038/srep42166

**Published:** 2017-02-10

**Authors:** Dom Bellini, Sam Horrell, Andrew Hutchin, Curtis W. Phippen, Richard W. Strange, Yuming Cai, Armin Wagner, Jeremy S. Webb, Ivo Tews, Martin A. Walsh

**Affiliations:** 1 Diamond Light Source, Harwell Science and Innovation Campus, Didcot, Oxfordshire, OX11 0FA, United Kingdom; 2 Research Complex at Harwell, Harwell Science and Innovation Campus, Didcot, Oxfordshire, OX11 0FA, United Kingdom; 3 School of Biological Sciences, University of Essex, Wivenhoe Park, Colchester, CO4 3SQ, Essex, United Kingdom; 4 Centre for Biological Sciences and Institute for Life Sciences, Life Sciences Building B85, The University of Southampton, University Rd, Southampton, Hampshire, SO17 1BJ, United Kingdom

## Abstract

The bacterial second messenger cyclic di-3′,5′-guanosine monophosphate (c-di-GMP) is a key regulator of bacterial motility and virulence. As high levels of c-di-GMP are associated with the biofilm lifestyle, c-di-GMP hydrolysing phosphodiesterases (PDEs) have been identified as key targets to aid development of novel strategies to treat chronic infection by exploiting biofilm dispersal. We have studied the EAL signature motif-containing phosphodiesterase domains from the *Pseudomonas aeruginosa* proteins PA3825 (PA3825^EAL^) and PA1727 (MucR^EAL^). Different dimerisation interfaces allow us to identify interface independent principles of enzyme regulation. Unlike previously characterised two-metal binding EAL-phosphodiesterases, PA3825^EAL^ in complex with pGpG provides a model for a third metal site. The third metal is positioned to stabilise the negative charge of the 5′-phosphate, and thus three metals could be required for catalysis in analogy to other nucleases. This newly uncovered variation in metal coordination may provide a further level of bacterial PDE regulation.

Cyclic di-3′,5′-guanosine monophosphate (c-di-GMP) is a universal second messenger in eubacteria that regulates a wide variety of functions including formation and dispersal of biofilms^1^,^2^,^3^,^4^, adhesion^1^,^5^,^6^, motility^7^,^8^,^9^, synthesis of virulence factors^10^ and developmental transitions^11^,^12^. Cellular levels of c-di-GMP are regulated by synthesis and degradation through diguanylate cyclases (DGCs) and phosphodiesterases (PDEs), respectively. DGC domains containing the signature GGDEF motif synthesise c-di-GMP from two GTP molecules^9^,^13^,^14^ while EAL^15^,^16^ and HD-GYP^17^,^18^,^19^ domain-containing PDEs are responsible for the hydrolysis of c-di-GMP to 5′-phosphoguanylyl-(3′-5′)-guanosine (pGpG). Structural and biochemical characterisation of GGDEF, EAL and HD-GYP domains provide a good understanding of the mechanisms of c-di-GMP turnover, interactions with other proteins and the direct and indirect effects of c-di-GMP levels on cell phenotype. A number of excellent reviews comprehensively summarise the current knowledge for c-di-GMP signalling in eubacteria and more specifically *Pseudomonas aeruginosa*^20^,^21^,^22^.

Bacteria within a biofilm are 100–1000 times more tolerant to antibiotics^23^,^24^,^25^. Therefore, being able to revert bacteria to a planktonic phenotype may present new opportunities to remove chronic infection^26^. As biofilm dispersal is associated with a reduction in intracellular levels of c-di-GMP, this strategy would likely depend upon c-di-GMP hydrolysing PDEs^3^,^5^,^9^. EAL domain containing proteins from a number of organisms have now been investigated structurally, with all EAL domains to date presenting with a highly conserved αβ(βα)_7-_barrel fold^27^,^28^,^29^. Most EAL domains possess PDE activity; although a significant group of inactive EAL domains exist, which likely function as c-di-GMP sensors^30^,^31^,^32^. Hydrolysis of c-di-GMP by EAL domains has been shown to require the presence of divalent ions, either Mg^2+^ or Mn^2+^, with the most efficient reactions occurring at alkaline pH^29^,^33^.

Dimerisation-induced conformational changes have been shown to regulate enzymatic activity in EAL-PDEs^33^,^34^,^35^,^36^. Specifically, dimerisation of the EAL domain YahA from *Escherichia Coli* has been shown to increase substrate-turnover, while specific site directed mutagenesis preventing dimerisation results in reduced PDE activity; without altering the substrate binding affinity^33^. This regulation is further rationalised through observations of EAL domain dimerisation in *P. aeruginosa* MorA, which causes the α5 helix to unwind at the dimerisation interface; allowing the catalytic DDFGTG motif on the β5-α5 loop (previously labelled as connecting β6 and α6^27^,^34^) to enter the substrate binding pocket and coordinate catalytically important metal ions in the active site^35^. Solution studies of *Klebsiella pneumoniae* BlrP1 identify this mechanism as regulatory in the context of full-length proteins, with the dimerisation interface and catalytic DDFGTG motif stabilised when activity is increased by stimulation of the regulatory BLUF domain^36^. Collectively these studies demonstrate how substrate-binding and metal coordination is controlled. Surprisingly, crystal structures of dimers have predominantly been reported with bound substrate, c-di-GMP^28^,^29^,^37^. This is not consistent with a model of activation through dimer formation and suggests that further regulatory steps must be required for the activation of EAL-PDEs.

A model organism for study of biofilm dispersal, and thus EAL-PDE behaviour, is *Pseudomonas aeruginosa* PAO1. The bacterial genome of this organism contains 41 proteins with PDE or DGC domains^38^,^39^, many of which regulate individual components of the biofilm phenotype^40^,^41^. As these seemingly redundant proteins have different roles related to biofilm behaviour we sought to determine conserved features of EAL-PDE activation.

Here we report as yet structurally uncharacterised EAL domains from the core genome of *Pseudomonas aeruginosa*, PA1727 (MucR) and PA3825. Structural analysis of MucR^EAL^ and PA3825^EAL^, in different metal binding states with or without substrate or product, provide evidence for the link between dimerisation and the formation of metal binding sites that are independent of the dimerisation interface. Finally we characterise a potential third metal binding site that directly interacts with the reaction product pGpG.

## Results

### Functional characterisation of MucR and PA3825 multi-domain proteins

We found that deletion of the PA1727 (*mucR*) gene leads to increased motility in *P. aeruginosa*; swim zones exhibited an average area of 0.762 ± 0.112 cm^2^ in the *∆mucR* strain compared to 0.499 ± 0.064 cm^2^ in the wild type (n = 6, P value = 0.001, 3 biological repeat experiments). MucR is a bifunctional transmembranous enzyme containing DGC and PDE activity that regulates alginate biosynthesis and biofilm dispersal^42^,^43^,^44^. Both cytoplasmic DGC and PDE domains are required to regulate the levels of c-di-GMP controlling alginate synthesis^43^,^45^. The *∆mucR* strain suggests that this multi-domain protein is an active DGC under test conditions, as deletion results in increased swimming motility, presumably due to a reduction in cellular c-di-GMP.

**Figure 1:**
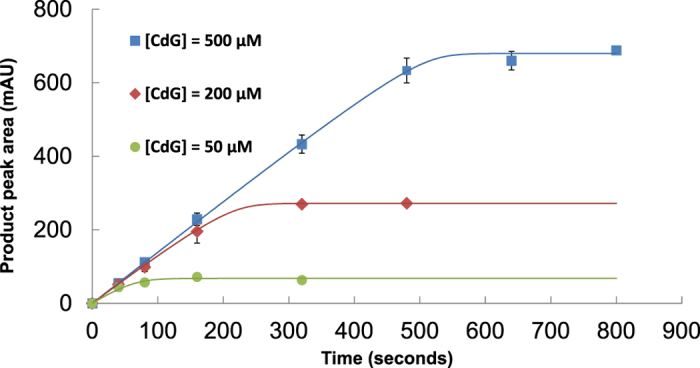
Kinetics analysis of PA3825^EAL^ in the presence of Mn^2+^. PA3825^EAL^ (3 μM final concentration) was added to different concentrations of c-di-GMP (labelled as CdG) and the progress of each reaction measured at specified time points through separation of nucleotides using Resource-Q FPLC, followed by integration of peaks at 254 nm absorbance.

Neither swimming, swarming, nor twitching motilities were affected in the PA3825 knockout strain under conditions tested. PA3825 is a two-domain cytoplasmic protein consisting of a structurally uncharacterised N-terminal CSS domain, with a predicted fold similar to the extracellular receptors of bacterial dimeric histidine kinases, and a PDE domain. However PA3825^EAL^ *in vitro* shows PDE activity in the presence of Mn^2+^, Fig. 1. *In vitro* PA3825^EAL^ was able to convert c-di-GMP to pGpG, with a *k*_*cat*_ of 0.35 ± 0.1 s^−1^, in the presence of Mn^2+^, consistent with observations of other active EAL-PDEs^15^,^29^,^33^.

### Different dimer contacts in c-di-GMP/Mg^2+^ bound MucR^EAL^ and PA3825^EAL^

**Figure 2:**
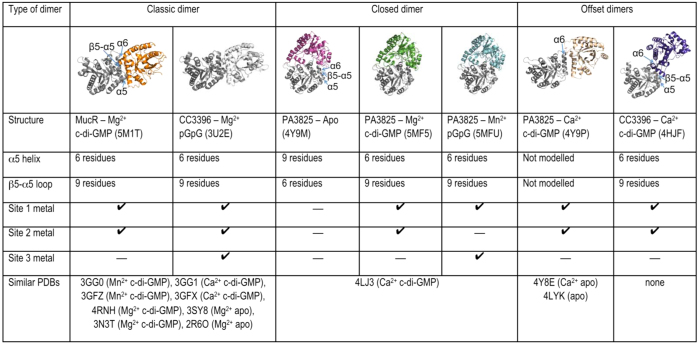
Analysis of dimerisation behaviour of EAL-PDE domains. Shown are different dimer architectures of EAL-PDEs, and associated structures identified in the PDB (as of November 2016). Information on the structure of the α5-helix and the β5-α5 loop is given; a short α5-helix (6 residues) is compatible with active site formation and metal binding through the two aspartate side chains of the DDFGTG motif on the elongated β5-α5 loop. Listed are the metal binding states and the nucleotide loading states.

We determined the PDE domain structures of MucR^EAL^ and PA3825^EAL^ in complex with c-di-GMP/Mg^2+^. Data processing and refinement statistics are reported in Table 1. Both PDE domains maintain the αβ(βα)_7_-barrel fold consistent with all EAL-PDE structures determined to date^27^. The MucR^EAL^ dimerisation interface involves helices α5 and α6 and has been observed in many other characterised PDE domains as listed in Fig. 2 (classic dimer). It is well established that significant structural rearrangements induced by dimer formation lead to a repositioning of active site residues to make the enzyme catalytically competent^33^,^35^,^36^.

**Figure 3:**
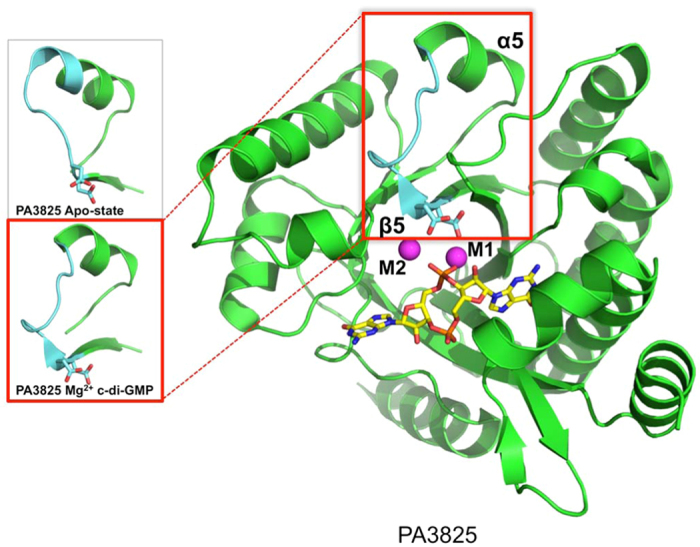
The structure of PA3825^EAL^ in complex with c-di-GMP/Mg^2+^. C-di-GMP is shown as yellow sticks and Mg^2+^ ions are represented by magenta spheres. Zoomed views of the β5-α5 loop, highlighted in light-blue, demonstrate the variation in the length of the α5 helix between the apo form and substrate forms of PA3825^EAL^. The structural shift in the β5-α5 loop allows Asp160 and Asp 161 to coordinate catalytic metals indicated as M1 and M2.

The structure of PA3825^EAL^ in complex with c-di-GMP/Mg^2+^ shows a different dimer interface to MucR, named here as the “closed dimer” (Fig. 2). We sought to confirm the dimerisation induced activation mechanism, seen in the classic dimer^33^,^34^,^36^, across this different dimer interface. The β5-α5 loop of PA3825^EAL^, residues 160–170, undergo significant changes between Mg^2+^-c-di-GMP bound and metal-free-apo states. The β5-α5 loop moves towards the active site and contributes to the formation of metal binding sites via two highly conserved Asp residues (Asp160–161, Fig. 3). The restructuring of the β5-α5 loop and the functional role in metal coordination is thus conserved across different dimer interfaces.

### The basis of EAL-PDE inhibition by Ca^2+^

**Figure 4:**
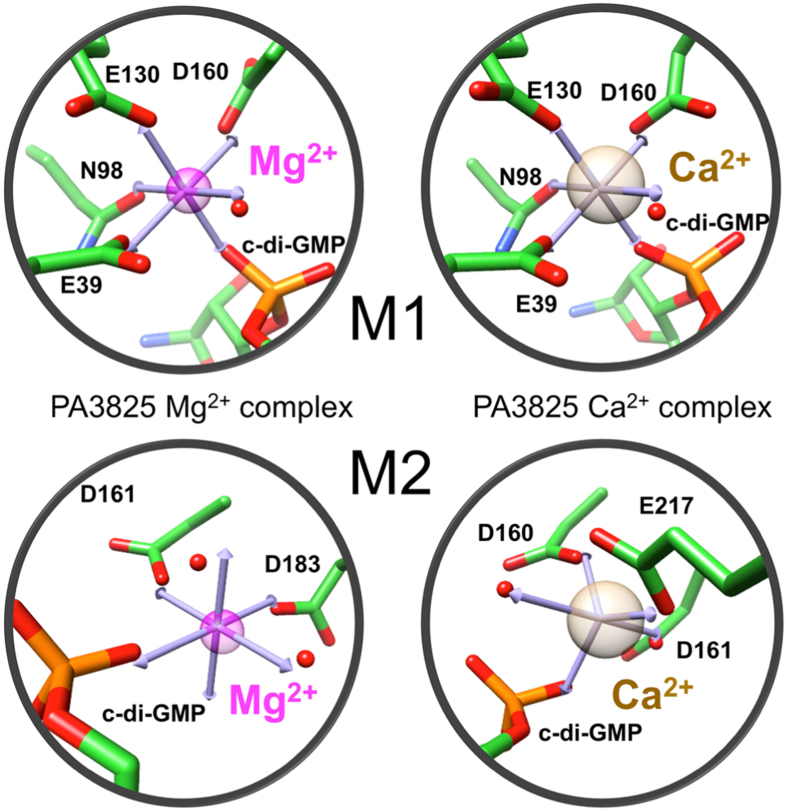
Perturbed metal geometry with altered activity in PA3825^EAL^. Idealised coordination geometries of the Mg^2+^- and Ca^2^-c-di-GMP PA3825^EAL^ complexes show the M1 metal ions coordinated in octahedral coordination. The M2 site metal in the Mg^2+^ -c-di-GMP PA3825^EAL^ complex is coordinated in an octahedral geometry, in comparison to the M2 metal site within the Ca^2+^ -c-di-GMP PA3825^EAL^ complex, which is coordinated in a trigonal bipyramidal geometry. Metal ions are shown as coloured transparent spheres (Mg^2+^ in magenta, Ca^2+^ in tan) with coordinating residues and nucleotide displayed in stick form with coordinating waters shown as red spheres.

Ca^2+^ is a well known inhibitor of EAL-PDE activity^15^,^16^,^29^. To better understand the reasons for the inhibitory role of Ca^2+^, we determined PA3825^EAL^ structures in the presence of Mg^2+^ or Ca^2+^ ions with bound c-di-GMP (Fig. 4). The Mg^2+^ and Ca^2+^ complexes show metals in the catalytic centre, annotated as M1 and M2, coordinating the phosphate group of the scissile phosphodiester in c-di-GMP.

The Mg^2+^ and Ca^2+^ metals in the M1 site superimpose well between the two complexes, (Fig. 4). In both cases, the metal is coordinated in an octahedral geometry by Glu39, Asn98, Asp130, Asp160, a non-bridging oxygen from the scissile phosphate (P1) of c-di-GMP and a putative in-line attacking water molecule (Fig. 4). Significant differences are seen in the positioning of the Asp160 side chain between metal bound complexes, with the second oxygen of Asp160 contacting a peptide nitrogen atom with Mg^2+^ bound; while the Ca^2+^ bound complex shows Asp160 bridging between the M1 and M2 metals.

The M2 metal shows octahedral coordination for Mg^2+^ but trigonal bipyramidal coordination geometry for Ca^2+^ (Fig. 4). Mg^2+^ is coordinated by Asp161, Asp183, a non-bridging phosphate (P1) oxygen and two waters (Fig. 4). This contrasts with the calcium complex, where Asp183 is not involved in coordination but instead Asp160 and Glu217 make contact to the Ca^2+^ in the M2 position. Both Asp160 and Asp161 are part of the conserved catalytic sequence motif **DD**FGTG. The different positioning of Asp160 and different coordination geometry of Ca^2+^, together with physicochemical properties, thus contribute to the inhibitory effect by calcium^46^,^47^.

### Structure of PA3825^EAL^ in complex with pGpG reveals a third metal binding site

**Figure 5:**
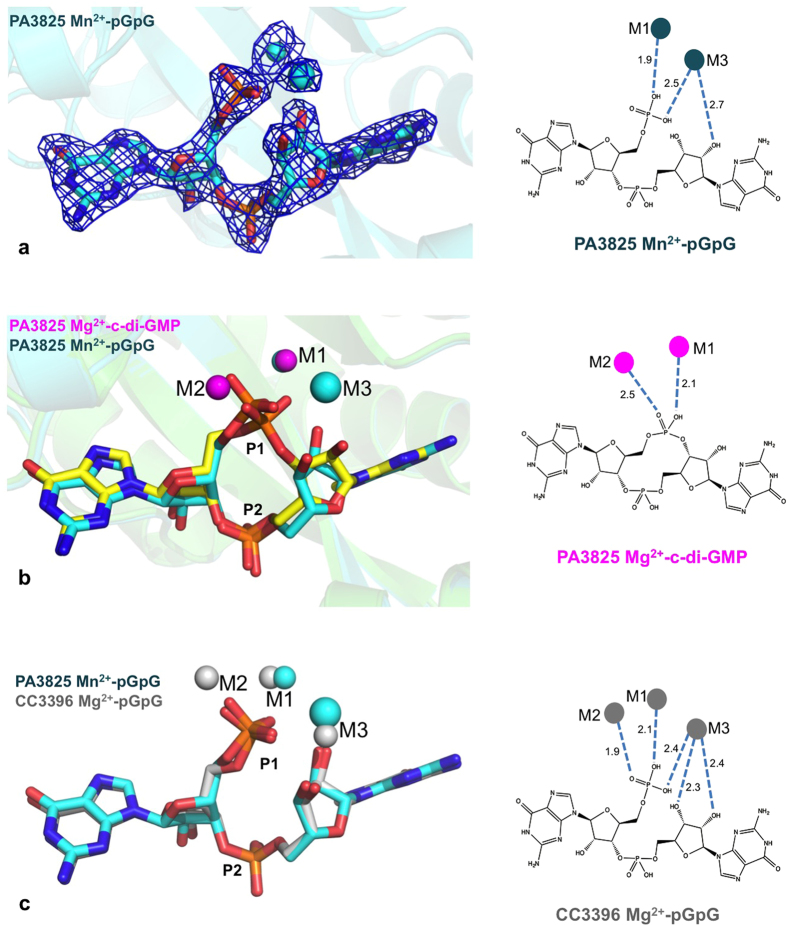
Identification of the M3 position in the pGpG complex. (**a**) Electron density of pGpG and metal ions bound to PA3825^EAL^, contoured at 1.3 σ. (**b**) Structural overlay of the substrate and product complexes of PA3825^EAL^. The substrate c-di-GMP is shown as yellow sticks; Mg^2+^ ions (magenta spheres) occupy the M1 and M2 binding sites. The product pGpG is shown as blue sticks; Mn^2+^ ions (blue spheres) occupy the M1 and M3 binding sites. (**c**) Structural comparison of the product complexes of PA3825^EAL^ and CC3396^EAL^ (PDB code 3U2E). In PA3825^EAL^ manganese and sodium ions are present in the M1 and M3 positions, respectively, while in CC3396^EAL^ magnesium ions are present in the M1, M2 and M3 positions. Schematic representations on the right show the interactions between the coordinated metal ions and the bound substrate or product, with distances labelled in Å.

Most PDB structures to date are reported either as apo-enzyme or as c-di-GMP substrate complexes, Table 2. We were fortuitous to observe PA3825^EAL^ with bound product pGpG, from co-crystallisation with c-di-GMP and Mn^2+^ (Fig. 5a). In this complex, the M1 site in the pGpG-bound structure is superimposable to the M1 metal in Mg^2+^ and Ca^2+^ complexes, the M2 site is vacant, but we observed a solvent binding site that interacts directly with the bound pGpG that presents metal like coordination geometry. We propose this as a potential third metal binding site, designated as M3.

Diffraction data collected above the manganese absorption edge confirmed the presence of Mn^2+^ in the M1 position in the pGpG/Mn^2+^ PA3825^EAL^ complex (Supplementary Fig S1) but showed no signal for manganese at the M3 metal like centre. Nonetheless, this metal-like M3 site is coordinated by the P1 phosphate non-bridging oxygen and the O2′ ribose oxygen (Fig. 5b). Consistent with the absence of any anomalous signal in the M3 site, we have placed and refined sodium in this location which is abundant in the crystallisation condition and consistent with the geometry observed^48^.

The M3 metal site displays a tetrahedral coordination sphere consisting of Asp 160, a water molecule, the ribose O2′ oxygen and a P1 hydroxyl oxygen from pGpG. To identify whether the M3 site is unique we searched the PDB and identified two EAL-pGpG complexes, Table 2. The first, FimX^49^ (PDB code 4AFY) is an inactive PDE and lacks the DDFGTG motif required for metal coordination^49^; the guanine bases in the FimX-pGpG complex are positioned in different positions from either the substrate complex or the product complexes of PA3825^EAL^, which in themselves superpose well. The second, CC3396^EAL^ from *Caulobacter crescentus* (PDB code 3U2E), shows pGpG adopting a near identical conformation to the pGpG moiety in PA3825^EAL^ (Fig. 5c). This structure also identifies a third metal binding site which aligns with the proposed M3 binding site in PA3825^EAL^. CC3396^EAL^ was refined and deposited with Mg^2+^ modelled at all three metal binding sites. These magnesium ions directly interact with the bound pGpG through coordination to oxygen atoms of the phosphate and the ribose around the scissile bond (Fig. 5c). In CC3396^EAL^ the M3 metal binding site coordination differs to that of PA3825^EAL^ in that the O3′ oxygen from the pGpG sugar ring and waters take the place of the conserved aspartate, allowing octahedral coordination of the d-metal Mg^2+^. In PA3825^EAL^ a tetrahedral coordination is observed for the s-metal Na^+^ complex. Superposition of the two complexes shows that the Na^+^ of PA3825^EAL^ is further removed from the scissile bond, compared with the Mg^2+^ observed in CC3396^EAL^, consistent with ionic radii and metal co-ordination geometries (Fig. 5c). The M1 and M2 metal sites of CC3396^EAL^ correspond to the characteristic M1 and M2 metal sites discussed above. In PA3825^EAL^, the M2 site is empty, but Glu217 (contributed by the β7-α7 loop in close vicinity to the β5-α5 loop) replaces interactions with the phosphate moiety normally made by M2. While PA3825^EAL^ lacks a metal in the M2 site and possesses one in the M3 site, the comparison with CC3396^EAL^, in which all three metal sites are occupied, suggests M3 site is a *bona fide* metal binding site in PA3825^EAL^.

## Discussion

**Figure 6:**
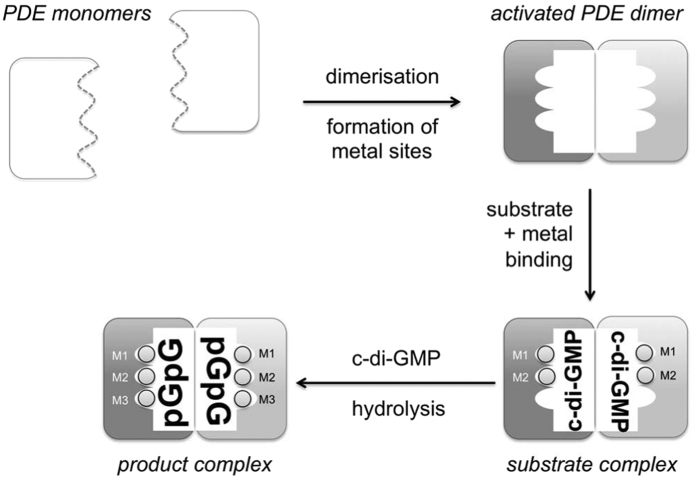
Proposed regulatory steps in EAL-PDE catalysed c-di-GMP hydrolysis. Dimerisation induces a conformational change in the β5-α5 loop, allowing conserved aspartate residues to coordinate metal ions in the M1 and M2 position and c-di-GMP to bind. A third metal ion binds in the M3 position in the pGpG product complex.

To identify features of EAL-PDE activation conserved across different phenotypic responses we have studied the EAL domains of the multi-domain proteins PA3825 and MucR from *P. aeruginosa*. While EAL-PDE activity is modulated by dimerisation through length variation of helix α5 and correct positioning of the metal coordinating motif DDFGTG^35^, we show conservation of this mechanism across different dimer interfaces (Fig. 2). The data presented also allows the identification of a third metal site M3 which is a hallmark of the product state. This may explain the observation of c-di-GMP substrate complexes despite apparent dimerisation and well-formed active sites with coordinated M1 and M2 metals. We summarise the structural changes that discriminate substrate and product bound states of EAL-PDEs in a scheme (Fig. 6).

The substrate- and product-bound forms of PA3825^EAL^ are characterised by changes around the α5 helix, consistent with an activation step from the apo/ground state induced by dimerisation. The model put forward requires dimerisation for activity that by itself would not be sufficient for gaining full activity. Summarising knowledge gained from structure determinations of different EAL PDEs suggests that binding of metal ions or ligands is always concurrent with a shortening of the α5-helix and linked to the requirement of protein-protein interactions (Fig. 2 and Table 2)^33^,^35^,^36^. Observation of the third metal binding site now requires rationalising.

Historically, mutagenesis and structural studies on RocR and YkuL led to catalytic proposals involving only a single-metal^27^,^28^. A number of c-di-GMP bound EAL structures with catalytic centres containing pairs of either Mg^2+^, Mn^2+^ or Ca^2+^ ions then lead to the proposals of two metal-ion catalysis^16^,^29^,^37^,^50^. Observed differences in inter-metal distances within a range of metal-substrate BlrP1 complexes at various pHs, with different *in vitro* activity, finally lead to the suggestion of metal ion migration within the active site regulating activity. It was proposed that metals move closer to one another to activate a water molecule for phosphodiester bond hydrolysis^29^. Further, deuterium exchange data showed that activation through a sensor domain (BLUF) and active site changes, including ordering of the α5 region, are consistent with preformed dimers^36^. These data together now point at further changes required in the active site for the catalytic cycle of phosphodiester bond hydrolysis.

Observation of the M3 metal site close to the hydrolysed phosphodiester bond in pGpG may now provide a first hint to rationalise the unexplained observations around enzyme activation. While metals in M1 and M2 are required for activation of the nucleophile, a metal in the M3 site is too far removed from the nucleophile to perform a similar function. Instead, the M3 metal is observed in the product state where it is ideally poised to stabilise the negatively charged transition state during hydrolysis of c-di-GMP to pGpG; where pGpG directly contributes to formation of the M3 metal site via its non-bridging phosphate oxygen (terminal phosphate group of pGpG). The M3 site is consistent with the pGpG-bound state of *Caulobacter crescentus* EAL PDE CC3396^EAL^ (PDB code 3U2E). The presence of a metal in the M3 site is concurrent with bound pGpG, providing a further example for requirement of a metal in this position.

Parallels exist between the proposed three-metal EAL-PDE and nuclease P1, along with other endonucleases. Binding of three Zn^2+^ ions has been shown in high-resolution structures for nuclease P1^51^ and endonuclease IV^52^,^53^. Quantum mechanics calculations since have supported functional roles for all three metals^54^,^55^, with the third metal ion positioned close to the hydrolysed bond to stabilise the negatively charged leaving group. The analogy holds for magnesium ions as well, as recent kinetic analysis of T5 flap endonuclease across a range of ion concentrations demonstrated the requirement for three magnesium ions in catalysis^56^. The EAL type PDEs thus might also require three metals in its catalytic cycle, which has parallels to a subset of HD-GYP type PDEs^19^,^57^.

## Conclusion

Understanding the regulation of PDE containing proteins is essential to study biofilm development and dispersal mechanisms. New structural data shows three metal sites in EAL-type phosphodiesterases. In the product complex, a metal directly interacts with the hydrolysed phosphodiester, and while the precise role of the newly identified M3 metal in catalysis requires further investigation, a role in stabilisation of the negative charge during catalysis is likely. We further describe a model of inhibition of EAL type PDEs using Ca^2+^, and demonstrate that variation of metal binding is fully consistent with the previously described enzyme activation mechanism by dimerisation, even involving different dimer interfaces. Understanding the regulatory layers of EAL-PDE activation may provide an angle to using PDEs as drug targets in biofilm dispersal.

### Experimental Procedures

#### Bacterial strains, media, and growth conditions

*Pseudomonas aeruginosa* and *Escherichia coli* strains used in this study are listed in Table S1^58^,^59^. All overnight cultures were routinely grown at 37 °C in LB medium. Antibiotics were used at the following concentrations: 30 μg/ml of gentamicin (Gm), 200 μg/ml of carbenicillin for *P. aeruginosa* and 15 μg/ml of Gm, 100 μg/ml of ampicillin for *E. coli S17-1*.

#### Construction of isogenic P. aeruginosa mutants

Isogenic mutants were constructed by replacing part of the *mucR* and PA3825 coding regions (as identified from the *Pseudomonas* genome database^60^) with a gentamicin (Gm) resistance cassette as previously described^61^. Briefly, two flanking up- and downstream regions (approximately 350–400 bp) from the *mucR* and PA3825 genes were amplified by standard PCR using primers listed in Table S2. Up- and downstream *mucR* and PA3825 DNA fragments were digested with EcoRI and HindIII and ligated to the Gm cassette (amplified from pPS856^59^) to give DNA fragments with the Gm cassette positioned between the up- and downstream regions of the gene of interest. The constructed fragments were then inserted into the SmaI site on pEX100 T, generating plasmids pEX100 T::*∆mucR*::Gm and pEX100 T::*∆*PA3825::Gm containing sacB genes for counter selection. Plasmids were introduced into *E. coli* S17-1 by chemical transformation and then transferred into PAO1 by conjugation. Transconjugants were first selected on *Pseudomonas* isolation agar containing 30 μg/ml Gm and then patched onto LB + 5% sucrose + 30 μg/ml Gm agar and LB + 200 μg/ml carbenicillin agar. Colonies that only grew on LB + 5% sucrose + 30 μg/ml Gm agar but not on LB + 200 μg/ml carbenicillin were selected as double-recombinant mutants. Mutants were confirmed by PCR, followed by sequencing.

#### Motility assays

To assess swarming motility, 3 μl overnight LB culture (absorbance at 600 nm, A_600_, 1.3–1.5) was inoculated onto 0.5% (w/v) agar with 8 g/L nutrient broth (Oxoid) and 5 g/L glucose (poured at 55 °C and dried under laminar flow for 40 mins) and incubated at 37 °C for 24 hrs.

To assess swimming motility, bacteria grown overnight on LB agar were inoculated onto the surface of a swimming plate (10 g/L tryptone (Oxoid)/5 g/L NaCl) containing 0.3% (w/v) agarose, poured at 55 °C and dried under laminar flow for 15 mins) with a sterile toothpick and incubated at 30 °C for 16–18 hrs.

To assess twitching motility, bacteria grown overnight on LB agar were stab inoculated into the bottom of 1% (w/v) LB agar (poured at 55 °C and dried under laminar flow for 1 hrs) with a sterile toothpick and incubated at 37 °C for 24 hrs.

#### Cloning, Expression and Purification

A DNA fragment for the EAL domain of PA3825 (residues 255–517) was PCR-amplified from the genome of *P. aeruginosa* strain PAO1 and subcloned into popinF vectors using in-fusion^TM^ cloning, and subsequently transformed into *E. coli* Lemo21 (DE3) cells in the presence of 50 μg/ml ampicillin. Cells were grown at 37 °C to an A_600_~0.6, induced with 0.15 mM IPTG and left at 18 °C for 16 h after induction. Cultures were subsequently spun at 5500 *g* for 10 minutes at 4 °C and the pellets resuspended in buffer A (0.1 M Tris-HCL pH 8.0, 0.5 M NaCl, 1 mM TCEP, 20 mM imidazole, 2% glycerol) plus 10 mM MgCl_2_, 10 μg/ml DNAse and 300 μg of lysozyme per gram of pellet. Cells were lysed by sonication in two cycles of 45 and 30 seconds and centrifuged for 15 minutes at 30,600 *g* at 4 °C. Cleared lysate was loaded onto a Ni-NTA affinity column pre-equilibrated in buffer A. Affinity chromatography was carried out in an AKTA purifier (GE Healthcare) according to the manufacturers’ instructions. The N-terminal His_6_-tag was cleaved overnight at 4 °C with a His_6_-tagged 3 C protease, which was subsequently removed by passing the sample back onto the Ni-NTA column. Fractions containing the untagged EAL domain were pooled together and concentrated to a final volume of 6 ml and loaded onto a Superdex 200 16/60 gel filtration column equilibrated with size exclusion chromatography (SEC) buffer (20 mM Tris-HCL, pH 8, 300 mM NaCl, 1 mM TCEP).

DNA coding for the EAL domain of MucR (residues 425–685) was also PCR–amplified from the genome of *P. aeruginosa* PAO1 and subcloned into pET-28a vector using HndIII and NdeI restriction sites (Novagen). This plasmid was then transformed into *E. coli* BL21 DE3 competent cells, grown in LB medium at 37 °C to an A_600_ of 0.6, before induction with 1 mM IPTG and incubated at 37 °C for a further 2 hours. Cultures were subsequently spun at 4,000 *g* for 20 mins at 4 °C and the pellets resuspended in buffer B (50 mM HEPES pH 7.5, 300 mM NaCl, 2 mM β-Mercaptoethanol and 5% glycerol). Cells were lysed by sonication for 5 minutes and centrifuged for 40 minutes at 30,000 *g*. The supernatant was loaded onto 4 ml of Ni-NTA resin (Qiagen), equilibrated with buffer B containing 20 mM imidazole, followed by washing with buffer B containing 80 mM imidazole and elution using buffer B containing 300 mM imidazole. The eluate was concentrated using spin-concentrators (Generon) before being loaded onto a S75 16/600 size exclusion column (GE) equilibrated in 50 mM HEPES pH 7.5, 300 mM NaCl, 2 mM MgCl_2_ and 2 mM β-Mercaptoethanol.

#### Enzymatic Assay

Enzymatic assays quantified the conversion of c-di-GMP to pGpG by ion exchange chromatography with a 1 ml Resource-Q column pre-equilibrated in 5 mM ammonium bicarbonate. Enzymatic reactions were carried out at room temperature as previously described^33^. PA3825^EAL^ was stripped of all metals with 50 mM EDTA, later removed by dialysis. Enzymatic assays were carried out in the presence of excess substrate and Mn^2+^ at 5 times excess. To monitor reaction progress, at different time points 100 μl of reaction solution was loaded onto the column and eluted over an 18 CV linear ammonium bicarbonate gradient (5 mM to 1 M). Elution was monitored by absorption at 254 nm. Nucleotide percentage was determined by integrating the nucleotide specific peaks. Global Kinetic Explorer software (KinTek, Austin, TX, USA) was used to fit the entire set of progress curves measured over a range of substrate concentrations.

#### Crystallisation and Data Collection

For all crystallisation experiments, PA3825^EAL^ was concentrated to 15–20 mg/ml (quantified by measuring absorbance at 280 nm, A_280_). Crystallisation was carried out by sitting drop vapour diffusion at a 1:1 protein/reservoir ratio. Primitive tetragonal crystals grew at 4 °C in 0.8 M sodium phosphate monobasic, 1.2 M potassium phosphate (dibasic) and 0.1 M sodium acetate pH 4.5 (overall pH of the final solution was found to be 7.1). Centred monoclinic crystals were grown at 4 °C in 6–11% isopropanol, 0.1 M MES pH 6.5, 0.1 M sodium acetate pH 4.5 and 0.2 M calcium acetate (overall pH 5.2). CaCl_2_, MgCl_2_ or MnCl_2_, respectively, were added at a concentration of 0.2 M to the crystallisation reservoir before drops were dispensed and 10 mM c-di-GMP was added directly to the protein sample to co-crystallise PA3825^EAL^ tetragonal crystals. In the case of metal-di-GMP ternary complexes, metal-loaded PA3825^EAL^ crystals, prepared as described above, were soaked with 10 mM c-di-GMP for 1 hour at 20 °C. The tetragonal crystal of PA3825^EAL^ containing the bound product pGpG were instead prepared by co-crystallisation of the protein with the substrate, by adding 10x excess of CdG to PA3825^EAL^ prior setting up crystallisation drops in the presence of MnCl_2_ (see above); under these conditions, only one crystal was obtained, which appeared after several weeks. All crystals were very briefly soaked in the appropriate crystallisation solution containing 25% ethylene glycol before flash freezing in liquid nitrogen. Single crystal diffraction datasets for metal-free-apo and metal-c-di-GMP complex crystals were collected at 100 K on beamlines I02 and I04-1 at Diamond Light Source, UK (Table 1).

MucR^EAL^ was concentrated to 13 mg/ml (quantified by measuring A_280_) and crystallised in 0.1 μL sitting drops. Crystals grew at 21 °C in 100 mM Morpheus Buffer System 1 pH 6.5, 120 mM Morpheus ethylene glycols and 37.5% of a MPD, PEG 1 K and PEG 3350 precipitant mix (Molecular Dimensions). Data were collected on I04 (Diamond Light Source) at 100 K (Table 1).

#### Structure Solution and Refinement

Diffraction datasets were processed with the *xia2* pipeline at Diamond with 5% of reflections removed for cross-validation^62^,^63^,^64^,^65^,^66^,^67^. All structures were solved by molecular replacement using either Molrep^68^ or MrBump^69^. Both MucR^EAL^ and the apo structure of PA3825^EAL^ were solved using residues 493–743 of the EAL domain from *Thiobacillus denitrificans* (PDB code 2R6O)^37^. PA3825^EAL^ metal-c-di-GMP and metal-pGpG complexes were solved using the refined apo structure as the search model. Further refinement of structures was performed with REFMAC5^70^, with model building carried out in COOT^71^. Model validation was performed using Molprobity^72^. Figures of all structures were prepared with the UCSF chimera package or PyMol version 1.3r1^71^,^72^,^73^,^74^.

## Additional Information

**How to cite this article:** Bellini, D. *et al*. Dimerisation induced formation of the active site and the identification of three metal sites in EAL-phosphodiesterases. *Sci. Rep.* **7**, 42166; doi: 10.1038/srep42166 (2017).

**Publisher's note:** Springer Nature remains neutral with regard to jurisdictional claims in published maps and institutional affiliations.

## Supplementary Material

Supplementary Information

## Figures and Tables

**Table 1:** Crystallographic data and refinement statistics.

Structure	PA3825^EAL^ Apo	PA3825^EAL^ Ca-c-di-GMP	PA3825^EAL^ Mg-c-di-GMP	PA3825^EAL^ Mn-pGpG	MucR^EAL^-Mg-c-di-GMP
Data Collection					
Space Group	P4_3_2_1_2	C2	P4_3_2_1_2	P4_3_2_1_2	P2_1_
Cell Axes (Å)	a = 64.3	a = 112.2	a = 64.6	a = 64.8	a = 46.4
	b = 64.3	b = 59.4	b = 64.6	b = 64.8	b = 116.1
	c = 134.0	c = 92.8	c = 135.7	c = 135.8	c = 52.1
Angles (°)	α = β = γ = 90	α = γ = 90	α = β = γ = 90	α = β = γ = 90	α = γ = 90
		β = 115.0			β = 102.5
Beamline	I03	I04-1	I02	I02	I04
Wavelength (Å)	0.9763	0.9200	0.9795	1.8785	0.9795
Resolution (Å)	64.34–1.60 (1.64–1.60)	51.29–2.44 (2.50–2.44)	29.16–1.77 (1.81–1.77)	27.16–2.15 (2.21–2.15)	45.27–2.27 (2.33–2.27)
Unique Reflections (#)	36008 (2598)	20405 (1494)	28763 (2072)	16288 (1689)	24883 (1831)
Measured Reflections (#)	373940 (26770)	91744 (7123)	178724 (13790)	61259 (6341)	170715 (12632)
Redundancy	9.3 (9.2)	4.5 (4.8)	6.2 (6.7)	3.8 (3.8)	6.9 (6.9)
R_pim_ (%)	3.1 (30.2)	2.6 (42.2)	2.2 (26.7)	7.2 (40.5)	6.9 (27.6)
I/σ (I)	18.0 (3.2)	17.3 (2.0)	18.8 (2.6)	6.6 (2.4)	11.3 (2.9)
Completeness (%)	100.0 (99.5)	98.1 (98.9)	99.5 (99.8)	98.8 (99.2)	99.9 (99.8)
Refinement					
Molecules/AU	1	2	1	1	2
R_work_/R_free_ (%)	18.3/23.2	23.8/28.5	19.9/23.2	20.2/26.1	19.5/23.8
Rmsd					
Bond Length (Å)	0.022	0.008	0.014	0.016	0.011
Bond Angles (°)	2.088	1.373	1.638	1.725	1.440
Average B Factor (Å^2^)					
Protein	21.4	61.0	32.9	36.8	36.6
Nucleotide	-	44.2	31.0	38.9	27.8
Metals	-	77.3	29.9	41.3	22.0
Water	28.9	53.1	35.4	37.7	36.0
Ramachandran (%)					
Favoured Regions	99.3	93.8	98.3	97.6	98.0
Allowed Regions	0.7	5.2	1.7	2.4	2.0
PDB Code	4Y9M	5MKG	5MF5	5MFU	5M1T

Values in parentheses refer to the highest resolution bin.

**Table 2:** Classification of all EAL domain structures in the PDB to date.

No metal C-di-GMP	No metal No substrate	1 metal (M1) No substrate	1 metal (M1) C-di-GMP	2 metals (M1 & M2) C-di-GMP	pGpG
**Monomeric**	**Monomeric**	**Dimeric**	**Dimeric**	**Dimeric**	**Dimeric**
*FimX-EAL (3 HV8*)	DosP-EAL (4HU3)	**Mg^2+^**	**Mg^2+^**	**Mn^2+^**	**Mn^2+^**
*FimX-EAL (4FOJ*)	*LapD-EAL (3PFM*)	TBD1265-EAL (2R6O)	MorA-EAL (4RNH)	Blrp1 (3GFZ)	PA3825-EAL (5MFU)
*FimX-EAL (4FOU*)	*FimX-EAL (3HV9*)	**Ca^2+^**	**Ca^2+^**	Blrp1 (3GG0)	**Mg^2+^**
*FimX-EAL (4FOK*)	CC3396-EAL (3S83)	PA3825-EAL (4Y8E)	Blrp1_B (3GFX)	**Mg^2+^**	CC3396-EAL
*FimX-EAL (4F3H*)			YkuI_AB (2W27)	PA3825-EAL (5MF5)	**no metal**
*FimX-EAL (4F48*)	**Dimeric**	**Tetrameric**		MucR-EAL (5M1T)	*FimX-EAL (4AFY*)
*LapD-EAL (3PJW*)	PA3825-EAL (4Y9M)	**Mg^2+^**		TBD1265_EAL (3N3T)	
*LapD-EAL (3PJX*)	YkuI (2BAS)	RocR (3SY8)		**Ca^2+^**	
*LapD-EAL (3PJU*)	*YdiV-EAL (3TLQ*)			PA3825-EAL (4Y9P)	
	Imo0131-EAL (4Q6J)			CC3396-EAL (4HJF)	
**Dimeric**	MorA-EAL (4RNJ)			Blrp1_A (3GFX)	
*LapD-EAL (3PJT*)	MorA-EAL (4RNI)			Blrp1 (3GG1)	
	*FimX-EAL (4AG0*)			YahA (4LJ3)	

Inactive EAL domains are indicated in italics.
