# Supplementary material for: Dimerisation induced formation of the active site and the identification of three metal sites in EAL-phosphodiesterases
Source: Sci Rep. 2017 Feb 10;7:42166. doi: 10.1038/srep42166 (PMC5301497; doi:10.1038/srep42166)
Supplement: Supplementary Information [file srep42166-s1.pdf]

SUPPLEMENT TO

Dimerisation induced formation of the active site and the identification of three metal sites in EAL-phosphodiesterases

Dom Bellini[a,b]*, Sam Horrell[a,c]*, Andrew Hutchin[a,d]*, Curtis W. Phippen[d], Richard W. Strange[c], Yuming Cai[d], Armin Wagner[a], Jeremy S. Webb[d], Ivo Tews[d], and Martin A. Walsh[a,b]

[a]Diamond Light Source, Harwell Science and Innovation Campus, Didcot, Oxfordshire, OX11 0DE, United Kingdom.

[b]Research Complex at Harwell, Harwell Science and Innovation Campus, Didcot, Oxfordshire, OX11 0DE, United Kingdom

[c]School of Biological Sciences, University of Essex, Wivenhoe Park, Colchester, CO4 3SQ, Essex, United Kingdom

[d]Centre for Biological Sciences and Institute for Life Sciences, Life Sciences Building B85, The University of Southampton, University Rd, Southampton, Hampshire, SO17 1BJ, United Kingdom

*Joint first authors

Corresponding authors:

Martin A. Walsh, E-mail: martin.walsh@diamond.ac.uk tel:+44 1235778518

Ivo Tews, E-mail: Ivo.Tews@soton.ac.uk tel:+44 2380594415

Table S1. Primers used in this study

Primer	Sequence (5'→3')
mucR deletion	
PA1727-up-F	ATGCCCCGGGCCATCCTGGCGTCCTACAC
PA1727-up-R	ATGCGAATTCAGCCACAGCGGGTCG
PA1727-dn-F	ATGCAAGCTTGCCTGGAGCCCAGCC
PA1727-dn-R	ATGCCCCGGGTCAGGCGACGCTGGC
pa3825 deletion	
PA3825-up-F	ATGCCCCGGGATTCCGGTGAAGCGAGC
PA3825-up-R	ATGCGAATTCCATCAGCAAGAGGCGTC
PA3825-dn-F	ATGCAAGCTTAATTGCATGCCCTGGG
PA3825-dn-R	ATGCCCCGGGAAGTGCGACGACGGAAG
Gm cassette	
Gm-F	ATGCGAATTCGAATTGACATAAGCCTGTTCGG
Gm-R	ATGCAAGCTTGAATTGGCCGCGG

Table S2. Strains and plasmids used in this study

Strains or plasmid	Genotype or phenotypes[a]	Source or reference
P.aeruginosa		
PAO1	Wild-Type	(Holloway, 1969)
Δ*MucR*::PAO1	*mucR*::Gm	This study
Δ*PA3825*::PAO1	*pa3825*::Gm	This study
E.coli		
S17-1(λpir)	TpR SmR recA, thi, pro, hsdR-M+RP4: 2-Tc:Mu: Km Tn7	Biomedal corp.
Plasmid		
pPS856	Ap^R, Gm^R; 0.83-kb blunt-ended SacI fragment from pUCGM	(Hoang *et al.*, 1998)
pEX100T	Ap^R; $oriT^+$ $sacB^+$ gene replacement vector	(Hoang *et al.*, 1998)

[a] Ap^R, ampicillin resistance; $sacB^+$, sucrose sensitive; Gm^R, gentamicin resistance

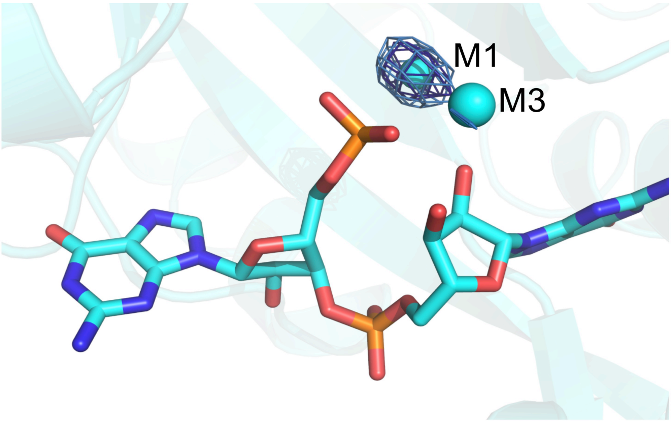

Fig. S1. Anomalous difference map of the PA3825^{EAL} pGpG/Mn^{2+} complex, displayed at 5.0 σ (dark blue) and 3.0 σ (light blue). Statistics displayed in Table 1.
